# Prospective, Observational Study of the Clinical Outcomes of FVIII Treatment in Adults and Adolescents with Severe Haemophilia A

**DOI:** 10.1055/a-2621-9749

**Published:** 2025-06-17

**Authors:** Pratima Chowdary, Liane Khoo, Michael Wang, Hervé Chambost, Anthony K.C. Chan, Annemieke Willemze, Johannes Oldenburg

**Affiliations:** 1 Katharine Dormandy Haemophilia and Thrombosis Centre, Royal Free Hospital, London, United Kingdom; 2 Department of Haematology, Cancer Institute, University College London, London, United Kingdom; 3 Haemophilia Treatment Centre, Royal Prince Alfred Hospital, Sydney, Australia; 4 Hemophilia and Thrombosis Center, University of Colorado Anschutz Medical Campus, Colorado, United States; 5 AP-HM, Department of Pediatric Hematology Oncology, Children Hospital La Timone & Aix Marseille University, INSERM, INRA, C2VN, Marseille, France; 6 Department of Pediatrics, McMaster Children's Hospital, McMaster University, Ontario, Canada; 7 Sanofi, Amsterdam, The Netherlands; 8 Institute of Experimental Hematology and Transfusion Medicine, University Hospital Bonn, Medical Faculty, University of Bonn, Bonn, Germany

**Keywords:** observational study, clinical study, haemophilia a, factor VIII, prospective studies

## Abstract

**Objective:**

To assess real-world treatment patterns and outcomes in previously treated patients ≥12 years old with severe haemophilia A treated with marketed factor VIII (FVIII) replacement products.

**Methods:**

Data were collected prospectively between 25 January 2019 and 30 November 2020 across 45 sites in 17 countries. Primary endpoint was annualized bleed rate (ABR). Secondary endpoints included factor consumption, bleed treatment, joint health, and safety. Exploratory endpoints included pain and quality of life outcomes.

**Results:**

A total of 157 patients received ≥1 FVIII injection (prophylaxis *n* = 139, on-demand *n* = 19). Mean (standard deviation; SD) observation period was 43.1 (13.3) weeks. Median (quarter [Q]1, Q3) ABR was 2.0 (0.0, 5.7) for those on prophylaxis. Those receiving standard half-life FVIII products or extended half-life FVIII products had a median (IQR) ABR of 2.2 (0.0, 6.1) and 1.3 (0.0, 5.0), respectively. Still, only 35% of patients on prophylaxis experienced zero bleeds and 18% had more than five bleeds in a year. Approximately 23% of bleeding episodes required >1 FVIII dose for resolution. The mean (SD) number of routine prophylaxis injections/week was 2.2 (1.1). Median (Q1, Q3) annualized factor consumption for patients on prophylaxis was 4,106.4 (3,151.6, 5,291.2) IU/kg/year. No changes in Haemophilia Joint Health Score (mean [SD] of 16.1 [19.3] versus 15.7 [17.7]), PROMIS pain intensity 3a T-score (mean [SD] 41.6 [8.2] versus 40.9 [9.1]), or Haem-A-QoL (mean [SD] 30.6 [17.3] versus 29.5 [17.4]) were observed between baseline and the end of the observation period for those using prophylaxis.

**Conclusions:**

Prophylaxis using standard or extended half-life FVIII replacement therapies achieves adequate haemostatic control in only about half of patients, with some experiencing very poor outcomes. Real-world data highlight the urgent need to optimize prophylaxis to enhance haemostatic control, ideally achieving a zero ABR and its associated benefits.

## Introduction

Regular prophylaxis initiated early in life is the standard of care (SoC) for people with severe haemophilia A, with the aim of preventing spontaneous bleeding episodes. This has been achieved either with replacement therapy using clotting factor concentrates or nonfactor therapy, including bispecific antibodies that act as activated factor VIII (FVIII) mimetics. 1 Historically, with clotting factor prophylaxis, physicians have targeted a FVIII trough of 1 IU/dL (1%), but guidelines from the World Federation of Hemophilia note that many physicians now target higher trough FVIII activity of 3 to 5 IU/dL or higher for their patients. 1

The introduction of extended half-life (EHL) factor-replacement therapies led to improved outcomes for patients compared with standard half-life (SHL) treatments and allowed patients to consistently maintain trough FVIII levels higher than 1%. 1 However, clinical and real-world evidence suggests bleeds continue to occur despite regular EHL prophylaxis, 2 3 4 5 even when higher trough levels of 8 to 12% are targeted. 6 Similarly, real-world evidence has shown breakthrough bleeding continues in patients treated with nonfactor therapy, 7 8 with a significant impact on patients, including repeated joint bleeds leading to chronic pain, impaired mobility, and the development of haemophilic arthropathy. 9 10 11 12 13

This study aimed to assess real-world outcomes in people with severe haemophilia A using marketed FVIII products, with the goal of increasing knowledge and understanding of the disease burden, treatment gaps, and unmet needs, in consideration of a rapidly changing haemophilia treatment landscape.

## Materials and Methods

### Study Design

This was a multicentre, prospective, observational study in which data were collected for ≤12 months from 25 January 2019 until 30 November 2020 (242HA201/OBS16221). Retrospective data including medical history and bleeding episodes were collected from patients' medical records for ≤12 months before study entry. Prospective data on joint health, treatment, bleeding episodes, patient-reported outcomes, FVIII genotype analysis, and healthcare resource utilization were recorded via electronic patient diary and electronic case report forms. This collection occurred at SoC visits, monthly telephone calls, and at study-specific visits at months 3, 6, and 12 ( Supplementary Fig. S1 ). Data were collected at 45 active sites across 17 countries to provide a broad geographic representation and reflect real-world treatment patterns worldwide ( Supplementary Table S1 ). The study, supported by Sanofi and Sobi, was performed in accordance with the Declaration of Helsinki and local regulations. Participating countries ensured necessary regulatory submissions were performed in accordance with local regulations including local data protection regulations. Investigative sites obtained written informed consent from participants.

As this was a prospective study designed to gather and summarize data, there was no planned statistical hypothesis testing. Patients could switch between on-demand and prophylactic treatment during the study, and if switched, their outcomes appeared in the summaries of both the on-demand and prophylactic groups. A sample size of approximately 150 subjects was targeted based on clinical considerations. Patients could withdraw early from the study to enter any subsequent Bioverativ (Sanofi)-sponsored study, with information from this observational study serving as baseline data for any subsequent study.

### Patient Population

The study enrolled adults and adolescents (≥12 years of age) with severe haemophilia A (<1 IU/dL [1%] FVIII activity, as per patients' medical records based on historical evidence from a certified clinical laboratory) previously treated (≥150 exposure days [EDs]) with any rFVIII or plasma-derived FVIII (pdFVIII) product (prophylaxis or on demand). Patients were required to be on a prophylactic or on-demand regimen with a marketed FVIII product at time of enrolment. If receiving on-demand treatment, patients were required to have had ≥12 bleeding episodes in the 12 months prior to study enrolment. Patients were excluded if they had any concurrent clinically significant major disease, other coagulation disorders, a history of a positive inhibitor test (≥0.6 BU/mL), or were in receipt of fitusiran or emicizumab. Patients with a family history of inhibitors were not excluded.

### Endpoints

The primary endpoint included spontaneous and traumatic annualized bleed rates (ABR). Secondary outcomes included annualized FVIII factor consumption, number of injections and dose to treat a bleed, and joint health as assessed using the Hemophilia Joint Health Score (HJHS). Safety endpoints included the incidence of treatment-emergent adverse events (TEAEs) and were assessed in the safety analysis set, which included only the subjects who received efmoroctocog alfa (prophylaxis or on demand; efmoroctocog alfa referred to herein as rFVIIIFc) at any point in the study. As this was a study funded by the co-developers of rFVIIIFc (Sanofi and Sobi), adverse events among those using rFVIIIFc were recorded.

Exploratory endpoints included Patient-Reported Outcomes Measurement Information System (PROMIS) Pain Intensity questionnaire and Haemophilia Quality of Life Questionnaire for Adults (Haem-A-QoL) in patients ≥18 years of age.

ABRs were calculated based on the total number of treated bleeding episodes during the observational period extrapolated to a 1-year interval of time. The observational period reflects the sum of all intervals of time during which patients were treated with an FVIII product within each treatment regimen, excluding surgical/rehabilitation periods and large injection intervals of >28 days. Model-based ABR values were estimated using a negative binomial model with the total number of treated bleeding episodes during the observational period as the response variable and log-transformed observational period. A bleeding episode was defined from the onset of the first sign of bleeding until ≤72 hours after the last injection to treat the bleeding episode.

HJHS version 2.1 was used to assess the health of six joints (left ankle, right ankle, left elbow, right elbow, left knee, and right knee) on a scale from 0 to 20. The following criteria were assessed: swelling, duration of swelling, muscle atrophy, crepitus of motion, flexion loss, extension loss, joint pain, and strength. Gait was scored on a scale from 0 to 4. The total score was the sum of the joint scores and gait score (total range from 0 to 124, with 0 being normal and 124 being the most severe disease); lower HJHS scores indicate better joint health outcomes. Assessments were made at baseline, months 3, 6, and 12, and at other clinic visits.

Haem-A-QoL consisted of 46 items across 10 dimensions: physical health (5 items), feelings (4 items), view of self (5 items), sports and leisure (5 items), work and school (4 items), dealing with haemophilia (3 items), treatment (8 items), future (5 items), family planning (4 items), and partnership and sexuality (3 items) administered to patients ≥18 years of age. Lower scores represent better quality of life (QoL). For children <18 years of age, the Haemo-QoL questionnaire was used; this instrument consists of 8 to 12 dimensions according to age group (with fewer items for younger children), covering domains of physical health (7 items), feeling (8 items), view of yourself (10 items), family (8 items), friends (4 items), others (6 items), sport and school (9 items), and treatment (8 items) among others. For both the Haem-A-QoL and Haemo-QoL, responses available included “never,” “rarely,” “sometimes,” “often,” or “all the time.”

QoL assessments were made at baseline, and months 3, 6, and 12. HJHS assessment was not performed at all visits for all patients, due to variations in standard practice at each site.

The PROMIS Pain Intensity instrument was used to assess pain intensity on a 5-point Likert scale. The tool consists of three questions on the patient's pain over the past 7 days, to which they were asked to respond: “had no pain,” “mild,” “moderate,” “severe,” or “very severe.” Raw scores are transformed into standardized T-scores. Lower PROMIS Pain Intensity 3a T-scores indicate better health outcomes. 14

The PROMIS Pediatric SF v2.0–Pain Interference 8a was used for participants <18 years of age. Eight statements related to how pain interfered with their activities over the last 7 days were rated by individuals as “never,” “almost never,” “sometimes,” “often,” or “almost always.” The PROMIS Pediatric SF v1.0–Physical Activity 8a assessment was used in those <18 years of age to gauge how physically active participants were in the prior 7 days. Responses included “no days,” “1 day,” 2 to 3 days,” “4 to 5 days,” or “6 to 7 days.” In both cases, raw scores were transformed into standardized T-scores. Lower PROMIS Pediatric SF Pain Interference 8a T scores indicate better health outcomes, while higher PROMIS Pediatric SF Physical Activity 8a T-scores indicate better health outcomes.

## Results

### Patient Disposition and Baseline Characteristics

A total of 158 patients were enrolled in the study. Of these, 157 males received ≥1 FVIII injection during the study and were included in the FAS (1 patient was excluded due to lack of treatment assignment). Among this 157, 139 received prophylactic treatment and 19 received on-demand treatment. One patient received on-demand and prophylactic treatment during the study and was included in both groups.

The mean (standard deviation [SD]) age of patients in the study was 32.9 (15.1) years. Patients were distributed across geographic regions in Europe (53%), North America (19%), the Asia/Pacific region (15%), and South America (13%). At enrolment, 35% of patients were receiving an EHL rFVIII treatment, 46% were receiving a SHL rFVIII treatment, 18% a pdFVIII treatment, and data was missing for 1%. The most common genotype was intron 22 inversion ( *n* = 32, 44%). Baseline demographics and haemophilia history are shown in Table 1 .

**Table 1:** Baseline demographics and haemophilia history

	Treatment regimen		
	Prophylaxis ( *n* = 139) a	On demand ( *n* = 19) a	Overall ( *N* = 157)
**Mean age, years (SD, range)**	32.7 (15.4, 12–69)	35.1 (13.0, 13–60)	32.9 (15.1, 12–69)
**Race,** ***n*** **(%)**			
*n* White Black or African American Asian Native Hawaiian or other Pacific Islander Not reported Other	137 85 (61.2) 6 (4.3) 22 (15.8) 1 (0.7) 16 (11.5) 7 (5.0)	19 14 (73.7) 0 0 0 2 (10.5) 3 (15.8)	155 98 (62.4) 6 (3.8) 22 (14.0) 1 (0.6) 18 (11.5) 10 (6.4)
**Region,** ***n*** **(%)** b			
Asia/Pacific Europe North America South America	24 (17.3) 78 (56.1) 23 (16.5) 14 (10.1)	0 6 (31.6) 6 (31.6) 7 (36.8)	24 (15.3) 83 (52.9) 29 (18.5) 21 (13.4)
**Family history of inhibitors,** ***n*** **(%)**			
Yes No Unknown	4 (2.9) 100 (71.9) 35 (25.2)	3 (15.8) 16 (84.2) 0	7 (4.5) 115 (73.2) 35 (22.3)
**FVIII genotype,** ***n*** **(%)**			
*n* Intron 22 inversion Intron 1 inversion Frameshift Missense Nonsense Other mutation Unknown	69 31 (44.9) 2 (2.9) 5 (7.2) 10 (14.5) 6 (8.7) 13 (18.8) 2 (2.9)	5 1 (20.0) 0 1 (20.0) 1 (20.0) 0 2 (40.0) 0	73 32 (43.8) 2 (2.7) 6 (8.2) 11 (15.1) 6 (8.2) 14 (19.2) 2 (2.7)
**Median number of bleeds in the past year (range)**	2.0 (0.0–120.0)	23.0 (12.0–68.0)	2.0 (0.0–120.0)
**Treatment at the time of study enrolment,** ***n*** **(%)**			
*n* EHL SHL or pdFVIII Unknown	138 54 (38.8) 82 (59.0) 2 (1)	19 1 (5.3) 18 (94.7) 0	157 55 (35.0) 100 (63.7) 2 (1) c
**HJHS**			
*n* Mean (SD)	91 16.1 (19.3)	11 26.2 (12.9)	102 17.2 (18.9)
HJHS category, *n* (%)			
0 to ≤10 >10 to ≤20 >20 to ≤30 >30	52 (57.1) 12 (13.2) 9 (9.9) 18 (19.8)	1 (9.1) 3 (27.3) 3 (27.3) 4 (36.4)	53 (52.0) 15 (14.7) 12 (11.8) 22 (21.6)

Abbreviations: EHL, extended half-life; FVIII, factor VIII; HJHS, Hemophilia Joint Health Score; pdFVIII, plasma-derived factor VIII; SD, standard deviation; SHL, standard half-life.

Notes: Percentages are based on the number of patients in the full analysis set.

a Patients may be counted in more than one category.

b The study enrolled across 17 countries: Argentina, Australia, Brazil, Bulgaria, Canada, Colombia, France, Germany, Greece, Hungary, Italy, Japan, Mexico, Netherlands, Taiwan, United Kingdom, and United States of America.

c Two patients did not have any FVIII classification at baseline and were not included in summary statistics for type of treatment received at baseline.

**Fig. 1:**
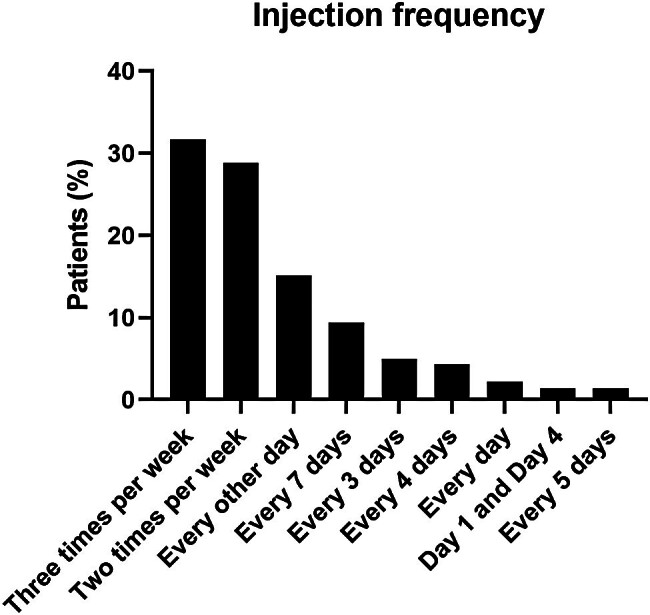
Injection frequency for the most recent prestudy prophylaxis regimen for patients in the prophylaxis arm ( *n* = 138). Percentages are based on the number of patients in the full analysis set.

The treatment frequency for patients' most recent prestudy regimen is shown in Fig. 1 for patients in the prophylaxis group. Most common were 3 times per week (44/139, 32%), 2 times per week (40/139, 29%), and every other day (21/139, 15%).

### Efficacy During the Observational Period

#### Bleed Rates

The overall mean (SD) observation period was 43.1 (13.3) weeks. The mean (SD) and median (quarter [Q]1, Q3) duration of the observational period was 42.3 (14.1) weeks and 48.4 (38.1, 52.1) weeks for prophylaxis, and 46.7 (8.4) weeks and 46.9 (43.3, 54.1) weeks for on-demand treatment. Overall, the mean (SD) exposure to FVIII treatment during the study was 97.0 (57.6) EDs (prophylaxis group: 104.9 [55.2] EDs; on-demand group: 33.8 [33.8] EDs).

There were 978 treated bleeding episodes during the study, 536 in the prophylaxis group (SHL or pdFVIII *n* = 410; EHL *n* = 120) and 442 in the on-demand group ( Table 2 ). During the study period, 91 patients (65%) in the prophylaxis group and all 19 patients in the on-demand group experienced ≥1 bleeding episode. The most common bleeding location was the joints in both the prophylaxis and on-demand groups (76 and 84% of bleeds, respectively), followed by muscle (16% in each group), skin/mucosa (9 and 5%), internal (4 and 3%), and unknown (1 and <1%).

**Table 2:** Bleeding outcomes a

	Treatment regimen			
	Prophylaxis ( *n* = 139)			On demand ( *n* = 19)
	SHL/pdFVIII ( *n* = 83) a	EHL ( *n* = 54) a	Overall ( *n* = 139)	
**Overall bleeds**				
*n* Median ABR (Q1, Q3) b Model-based mean ABR (95% CI) b c Patients with zero bleeds, *n* (%)	410 2.2 (0.0, 6.1) 6.0 (4.2–8.5) 28 (33.7)	120 1.3 (0.0, 5.0) 3.0 (2.1–4.4) 20 (37.0)	536 2.0 (0.0, 5.7) 4.8 (3.7–6.2) 48 (34.5)	442 29.1 (8.4, 40.0) 25.3 (17.1–37.4) 0
ABR distribution (%)				
0 to <2 2 to <5 5 to ≤10 >10 to ≤20 >20	40 (48.2) 18 (21.7) 11 (13.3) 9 (10.8) 5 (6.0)	30 (55.6) 11 (20.4) 8 (14.8) 5 (9.3) 0	70 (50.4) 31 (22.3) 19 (13.7) 14 (10.1) 5 (3.6)	2 (10.5) 1 (5.3) 2 (10.5) 3 (15.8) 11 (57.9)
**Spontaneous bleeds**				
*n* Median ABR (Q1, Q3) b Model-based mean ABR (95% CI) b c Patients with zero bleeds, *n* (%)	263 1.0 (0.0, 3.9) 3.7 (2.4–5.5) 40 (48.2)	50 0.0 (0.0, 1.7) 1.3 (0.7–2.3) 37 (68.5)	316 0.0 (0.0, 3.0) 2.7 (1.9–3.8) 78 (56.1)	331 14.7 (7.7, 30.1) 18.9 (12.5–28.6) 1 (5.3)
ABR distribution (%)				
0 to <2 2 to <5 5 to ≤10 >10 to ≤20 >20	51 (61.4) 19 (22.9) 9 (10.8) 2 (2.4) 2 (2.4)	44 (81.5) 5 (9.3) 5 (9.3) 0 0	96 (69.1) 25 (18.0) 14 (10.1) 2 (1.4) 2 (1.4)	3 (15.8) 1 (5.3) 2 (10.5) 4 (21.1) 9 (47.4)
**Joint bleeds**				
*n* Median ABR (Q1, Q3) a Model-based mean ABR (95% CI) b c Patients with zero bleeds, *n* (%)	326 1.2 (0.0, 4.1) 4.8 (3.2–7.2) 35 (42.2)	74 0.0 (0.0, 2.9) 2.0 (1.2–3.1) 29 (53.7)	406 1.1 (0.0, 3.7) 3.7 (2.7–5.0) 64 (46.0)	371 20.4 (7.2, 34.5) 21.1 (13.6–32.7) 1 (5.3)
ABR distribution (%)				
0 to <2 2 to <5 5 to ≤10 >10 to ≤20 >20	47 (56.6) 18 (21.7) 10 (12.0) 4 (4.8) 4 (4.8)	34 (63.0) 10 (18.5) 9 (16.7) 1 (1.9) 0	81 (58.3) 30 (21.6) 19 (13.7) 5 (3.6) 4 (2.9)	3 (15.8) 1 (5.3) 2 (10.5) 2 (10.5) 11 (57.9)
**Traumatic bleeds**				
Bleeds, *n* Median ABR (Q1, Q3) b Model-based mean ABR (95% CI) b c Patients with zero bleeds, *n* (%)	135 1.0 (0.0, 2.1) 2.0 (1.4–2.9) 40 (48.2)	62 0.0 (0.0, 2.1) 1.5 (1.0–2.4) 28 (51.9)	200 1.0 (0.0, 2.1) 1.8 (1.4–2.4) 68 (48.9)	103 4.8 (0.0, 8.7) 5.9 (3.3–10.5) 5 (26.3)
ABR distribution (%)				
0 to <2 2 to <5 5 to ≤10 >10 to ≤20 >20	59 (71.1) 12 (14.5) 9 (10.8) 2 (2.4) 1 (1.2)	38 (70.4) 10 (18.5) 5 (9.3) 1 (1.9) 0	98 (70.5) 23 (16.5) 14 (10.1) 3 (2.2) 1 (0.7)	8 (42.1) 2 (10.5) 6 (31.6) 2 (10.5) 1 (5.3)
**Bleed locations,** ***n*** **(%)**				
*n* Internal Joint Muscle Skin/Mucosa Unknown	410 14 (3.4) 326 (79.5) 61 (14.9) 31 (7.6) 6 (1.5)	120 8 (6.7) 74 (61.7) 27 (22.5) 19 (15.8) 1 (0.8)	536 22 (4.1) 406 (75.7) 88 (16.4) 50 (9.3) 7 (1.3)	442 13 (2.9) 371 (83.9) 69 (15.6) 21 (4.8) 1 (0.2)

Abbreviations: ABR, annualized bleed rate; CI, confidence interval; FVIII, factor VIII; Q, quarter; SD, standard deviation.

Notes: Data are based on treated bleeding episodes. Percentages of patients in each treatment regimen with an evaluable observational period.

a Bleed data from two patients were not included as the patients were not classified as having received SHL/pdFVIII or EHL therapy.

b ABRs were calculated based on the total number of bleeding episodes during the observational period extrapolated to a 1-year interval of time. The observational period reflects the sum of all intervals of time during which patients were treated with a currently marketed FVIII product within each treatment regimen, excluding surgical/rehabilitation periods and large injection intervals (>28 days).

c Estimated using a negative binomial model with the total number of treated bleeding episodes during the observational period as the response variable and log-transformed observational period.

The median (Q1, Q3) ABR was 2.0 (0.0, 5.7) and 29.1 (8.4, 40.0) in the prophylaxis and on-demand groups, respectively. The mean model-based (95% confidence interval [CI]) ABR was 4.8 (3.7–6.2) in the prophylaxis group and 25.3 (17.1–37.4) for on-demand group. For patients on prophylaxis, mean model-based mean (95% CI) was 6.0 (4.2–8.5) for those who received SHL or pdFVIII and 3.0 (2.1–4.4) for those who received EHL. Model-based mean (95% CI) spontaneous ABRs were 2.7 (1.9–3.8) in the prophylaxis group and 18.9 (12.5–28.6) in the on-demand group, while model-based mean joint ABRs were 3.7 (2.7–5.0) and 21.1 (13.6–32.7), respectively. Additional bleed rates stratified by type and location are shown in Table 2 .

There were more patients in the prophylaxis group who experienced overall ≤1, spontaneous, and joint bleeds than in the on-demand group. Conversely, patients in the on-demand group experienced >20 bleeding episodes more frequently for all three bleed outcomes ( Table 2 ). Notably, 35% of patients in the prophylaxis group experienced zero bleeds.

#### Bleed Treatment

The median (Q1, Q3) number of injections required to resolve a bleeding episode was 1 (1, 1), regardless of treatment type (prophylaxis or on demand; Supplementary Table S2 ). The mean (SD) number of injections required to resolve a bleeding episode was 1.9 (5.1) in the prophylaxis group and 1.3 (0.6) in the on-demand group. Consistent with this, 79% of bleeding episodes across both groups were resolved with a single dose of FVIII (prophylaxis: 77%; on demand: 81%). The median (Q1, Q3) number of injections required to resolve a bleeding episode was similar for patients in the prophylaxis group who received an EHL and those who received an SHL or pdFVIII (1.0 [1.0, 1.0] vs. 1.0 [1.0, 1.0]).

Overall, the median (Q1, Q3) total dose required to treat a bleeding episode was 30.2 (23.3, 40.7) IU/kg. In the prophylaxis and on-demand groups, the median (Q1, Q3) total dose required to treat a bleeding episode was 37.1 (26.4, 50.8) IU/kg and 26.3 (17.0, 30.3) IU/kg, respectively.

##### Factor Consumption

Factor consumption was higher in the prophylaxis group, with a median (Q1, Q3) annualized factor consumption of 4,106.4 (3,151.6, 5,291.2) IU/kg/year in the prophylaxis group and 875.0 (564.1, 1,049.1) IU/kg/year in the on-demand group. Median (Q1, Q3) weekly consumption (IU/kg/week) for those on prophylaxis was 78.7 (60.4, 101.4) IU/kg, whereas those on demand had a median (Q1, Q3) weekly consumption of 16.8 (10.8, 20.1) IU/kg.

For patients on prophylaxis, median (Q1, Q3) factor consumption was higher for those treated with SHL (4,386.5 IU/kg/year [3,220.1, 6,238.4]) than EHL (3,910.4 IU/kg/year [3,109.9, 4,750.8]) or pdFVIII (3,541.8 IU/kg/year [2,655.3, 5,599.3]). The median (Q1, Q3) weekly consumption for those using SHL prophylaxis (84.1 [61.7, 119.6] IU/kg/week) was higher than for those using EHL (74.9 [59.6, 91.1] IU/kg/week) or pdFVIII (67.9 [50.9, 107.3] IU/kg/week) prophylaxis.

The mean (SD) number of routine prophylaxis injections per week was 2.2 (1.1) overall, and 2.8 (1.4), 1.9 (0.70), and 2.6 (1.70) in patients who received SHL, EHL, or pdFVIII, respectively.

##### Joint Health, Pain, and QoL Outcomes

**Fig. 2:**
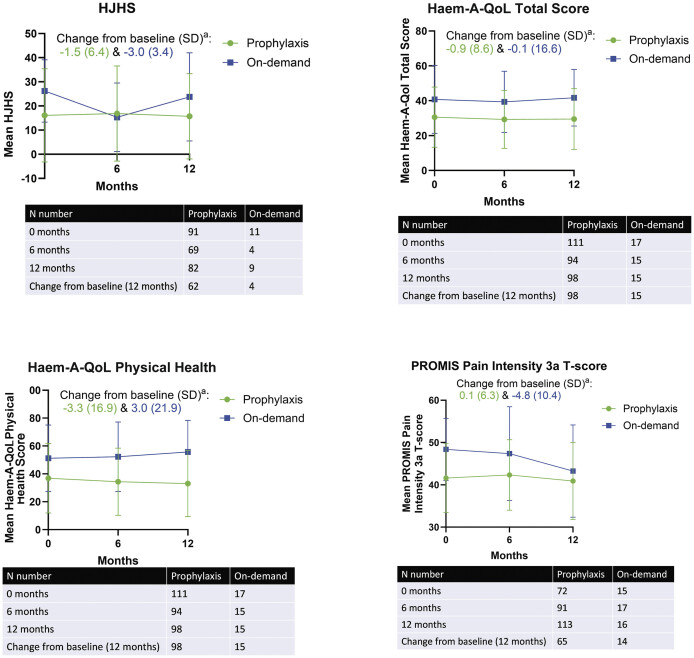
Joint health, quality of life (QoL), and pain outcomes. ^a^ Subscale scores and total score are presented as Transformed Scale Scores (TSS) ranging from 0 to 100%. Lower scores indicate better health outcomes. Haem-A-QoL, Haemophilia Quality of Life Questionnaire for Adults; HJHS, Hemophilia Joint Health Score; PROMIS, Patient-Reported Outcomes Measurement Information System; SD, standard deviation.

At baseline, mean HJHS was lower in the prophylaxis group than the on-demand group (16.1 [19.3] vs. 26.2 [12.9]; Table 1 ), suggesting better joint health in patients receiving prophylactic than on-demand treatment. However, even in patients receiving prophylaxis, extensive joint damage was noted, and this is related to the multinational nature of the study. Moreover, data on the duration of prophylaxis before study entry was not collected. HJHS remained relatively consistent over the study, with a change from baseline to 12 months (SD) of −1.5 (6.4; *n* = 62) and −3.0 (3.4; *n* = 4) for patients treated with prophylaxis and on demand, respectively ( Fig. 2 ).

Similar trends were seen for QoL, as assessed by Haem-A-QoL total score and Haem-A-QoL physical health score, and pain, as assessed by PROMIS Pain Intensity 3a T-score ( Fig. 2 ). Haemo-QoL and PROMIS Pain Interference 8a and Physical Activity 8a scores were relatively stable over time for children and adolescents on prophylaxis ( Supplementary Fig. S2 ).

##### Immunogenicity and Safety

There was a total of 58 TEAEs among the 51 patients who received rFVIIIFc (prophylaxis or on demand) at any point in the study and were assessed for safety. All TEAEs occurred within the prophylaxis group ( *n* = 50), including 3 TEAEs in 2 patients during a major surgery period ( Supplementary Table S3 ). In all 29 patients (58%) experienced ≥1 TEAE, and 5 (10%) experienced ≥1 serious TEAE. One patient experienced a TEAE with fatal outcome: post-procedural haemorrhage following removal of a duodenal tumour. None of the AEs reported were related to FVIII-replacement therapy, as assessed by investigators. There were no reports of inhibitor development to FVIII or anaphylaxis following treatment administration during the study.

## Discussion

This prospective observational study provides insight into treatment patterns in a population of people with severe haemophilia A across 17 countries. Most patients (approximately 90%) received prophylactic treatment with factor replacement, with approximately a third receiving an EHL; patients on a bispecific antibody could not enrol into the study. Although participants who received prophylactic EHL treatment had lower ABRs than those receiving SHL FVIII or pdFVIII prophylaxis, overall, those using prophylaxis still experienced suboptimal bleed control, with an overall model-based mean ABR of 4.8, a modeled mean joint ABR of 3.7, and with 49% experiencing ≥2 bleeds during the observation period. Additionally, this study shows that there is still a fraction of people with haemophilia who remain on on-demand regimens, which contributes to poorer outcomes, as evidenced by poorer joint health and QoL scores than those on prophylaxis.

Although most participants in this study were receiving prophylaxis against bleeding, only 35% of patients in the prophylaxis group experienced zero bleeds. This is consistent with clinical and real-world evidence showing patients with severe haemophilia A continue to experience bleeds despite receiving FVIII prophylaxis. 4 6 15 16 17 18 19 20 21 Studies of patients using EHL factor replacements indicate individuals may continue to experience bleeds; recently, one small assessment of adolescents using damoctocog alfa pegol showed 30% of patients experienced bleeds within a 12-month period (overall median total ABR was 1.8 across the main and extension study). 22 Another recent non-interventional study of males with haemophilia using either EHL or SHL prophylaxis indicates that though bleeding outcomes were better for individuals utilizing EHL FVIII as compared with SHL FVIII, the mean ABRs were still 1.5 and 2.3, respectively. 23

A retrospective review across several products, including EHL and SHL FVIII, shows that the proportion of patients who experience zero bleeds are higher for EHL FVIII (up to 62%), as compared with SHL FVIII prophylaxis, where the proportions were approximately 45%. 24 Additionally, in a US chart review that included 240 people with haemophilia, ABRs ranged from 2.5 to 4.8 across 6 EHL products. 3 Mean ABR outcomes in a US chart review of 120 people with haemophilia were 2.0 to 3.2 and 3.2 to 7.2 in patients treated 2 and 3 times per week with one of three different EHLs, respectively. 5 Overall, clinical trials and real-world evidence indicate that despite receiving prophylactic FVIII replacement therapy, a significant proportion of patients may still experience bleeding.

The impact of even small numbers of bleeds per year can be significant, with evidence that a single joint bleed may lead to persistent synovial inflammation. 25 26 27 Repeated bleeding results in joint health deterioration, chronic pain, 28 29 restricted mobility, 28 30 and impaired QoL. 31 Improving treatment outcomes may require targeting normal or near-normal factor levels. 32 This is supported by regression models, in which FVIII levels of up to 35% were required to achieve near-zero joint bleed rates. 33 34 35 In this study, 42% of patients on prophylaxis experienced ≥2 joint bleeds. HJHS remained stable over the relatively short study follow-up time. Long-term data have shown a decline in joint health outcomes for patients with severe haemophilia A, despite the administration of early prophylaxis. 9

It is possible to transiently achieve high FVIII levels with SHLs, while the development of EHL treatments has extended the time over which higher levels can be maintained. However, EHLs are limited by the von Willebrand factor (VWF)-imposed half-life ceiling, which constrains the half-life of factor replacement therapies. 15 36 37 38 39 Consistent with this, 84% of patients in this study were administered multiple routine prophylaxis injections per week, with approximately one-third dosing 3 times per week. Patients on prophylaxis demonstrated higher annualized factor consumption and better outcomes than patients in the on-demand group over the course of the study.

Approaches have emerged that offer an alternative to standard or extended half-life factor-based treatment. For example, emicizumab is a nonfactor therapy and as such is not directly affected by the VWF half-life ceiling although additional haemostatic support may be required. 7 8 40 41 42 43 44 45 Gene therapy holds the promise of a one-time treatment that could reduce the burden of administration, but concerns remain over FVIII levels over time, 46 interpatient variability, 47 48 and accessibility/cost. 49 The development of new treatments, including those able to overcome the VWF half-life ceiling 50 may may help negate the requirement of multiple injections per week.

The reported TEAEs with rFVIIIFc were generally consistent with those anticipated 51 in adult/adolescents with severe haemophilia A and no new safety concerns were identified.

This being an observational study, treatment was chosen by patients and treating physicians in the cohort presented here, which can lead to confounding variables affecting results observed. The frequency of assessments and ability to perform blood tests at similar, regular intervals due to the observational nature of the study also limited the data available. Moreover, the high prevalence of joint damage reflects the lifetime exposure to prophylaxis, including age of initiation and intensity of prophylaxis. Although there are some inherent limitations of an observational study, the strength of a study of this nature is that the results reflect real-world outcomes for people with haemophilia under their usual care.

## Conclusion

It has been proposed that the ultimate goal for people with haemophilia A should be life equity, whereby individuals live a life free from bleeding and with levels of mobility that allow them to enjoy a fulfilling and active life. 32 Currently, achievement of this goal is prevented by limits to the haemostasis that can be achieved with most common treatments. 32 The results of this study support this, showing that despite being well-treated with FVIII prophylaxis, bleeding is not necessarily well controlled and patients still suffer unacceptable deficits to their joint health, pain, and QoL. These outcomes were observed despite a high burden of treatment, with the majority of patients receiving multiple routine prophylaxis injections per week.

Overall, these data support the need for a new treatment paradigm, in which higher levels of protection are targeted to reduce bleed rates and improve outcomes, including joint health, QoL, and pain for people with severe haemophilia A.

## Notes

**Corrigendum:** This article has been corrected as per the corrigendum published online on August 21, 2025 (doi:10.1055/a-2685-5492).
