# Corrigendum: Prospective, Observational Study of the Clinical Outcomes of FVIII Treatment in Adults and Adolescents with Severe Haemophilia A

**DOI:** 10.1055/a-2685-5492

**Published:** 2025-08-21

**Authors:** Pratima Chowdary, Liane Khoo, Michael Wang, Hervé Chambost, Anthony K.C. Chan, Annemieke Willemze, Johannes Oldenburg

**Affiliations:** 1 Katharine Dormandy Haemophilia and Thrombosis Centre, Royal Free Hospital, London, United Kingdom; 2 Department of Haematology, Cancer Institute, University College London, London, United Kingdom; 3 Haemophilia Treatment Centre, Royal Prince Alfred Hospital, Sydney, Australia; 4 Hemophilia and Thrombosis Center, University of Colorado Anschutz Medical Campus, Colorado, United States; 5 AP-HM, Department of Pediatric Hematology Oncology, Children Hospital La Timone & Aix Marseille University, INSERM, INRA, C2VN, Marseille, France; 6 Department of Pediatrics, McMaster Children's Hospital, McMaster University, Ontario, Canada; 7 Sanofi, Amsterdam, The Netherlands; 8 Institute of Experimental Hematology and Transfusion Medicine, University Hospital Bonn, Medical Faculty, University of Bonn, Bonn, Germany

It has been brought to the publisher's attention that a typographical error appeared in the abstract of the article published in *TH Open* , Volume 9, a26219749, on June 17, 2025 (doi:  ). The abstract incorrectly stated that 18% of patients on prophylaxis had “five or more” bleeds, whereas it should have stated “more than five.” In other words, 18% of patients experienced more than five bleeds in a year while on prophylaxis. The abstract has now been corrected to reflect the accurate information.

