# Supplementary material for: Prospective, Observational Study of the Clinical Outcomes of FVIII Treatment in Adults and Adolescents with Severe Haemophilia A
Source: TH Open. 2025 Jun 17;9:a26219749. doi: 10.1055/a-2621-9749 (PMC12223957; doi:10.1055/a-2621-9749)
Supplement: Supplementary file 1 — Supplementary Material [file 10-1055-a-2621-9749_26872874.pdf]

Supplementary Table S1 List of enrolling sites and principal investigators

Country	Enrolling site	Principal investigator
**Argentina**	Dr Alfredo Pavlovsky Care Institute, Buenos Aires	Dr Daniela Neme
	William Osler Institute, Buenos Aires	Dr Patricia do Nascimento
**Australia**	Fiona Stanley Hospital, Perth	Dr Stephanie P'Ng
	Royal Prince Alfred Hospital, Institute of Haematology, Sydney	Dr Liane Khoo
**Brazil**	Campinas University Clinical Hospital, Hematology and Hemotherapy Center (HEMOCAMP), Sao Paulo	Dr Margareth Ozelo
**Bulgaria**	University Multiprofile Hospital for Active Treatment "Sveti Georgi," Plovdiv, Clinic of Clinical Hematology, Plovdiv	Dr Emil Spasov
**Canada**	McMaster Children's Hospital, Ontario	Dr Anthony K.C. Chan
	Montreal Children's Hospital, Quebec	Dr Catherine Vezina
**Colombia**	Pablo Tobon Uribe Hospital, Antioquia	Dr Kenny Galvez Cardenas
	De La Costa Clinic Ltd., Atlantico	Dr Fausto Vitali
**France**	Morvan Hospital, Department of Hematology, Finistere	Dr Brigitte Pan-Petesch
	Louis Pradel Hospital, Lyon University Hospital Center, Department of Biological Hematology and Clinical Hemostasis, Lyon	Dr Claude Negrier
	Cardiology Hospital, Hemorrhagic Pathology, Lille	Dr Sophie Susen
	Timone University Hospital, Department of Pediatric Hematology, Marseille	Dr Hervé Chambost
**Germany**	University Hospital Bonn, Institute of Experimental Hematology and Transfusion Medicine, Bonn	Dr Johannes Oldenburg
	Johann Wolfgang Goethe University Hospital, Center of Pediatric Oncology, Hematology and Hemostaseology, Frankfurt-am-Main	Dr Christoph Königs
	Vivantes Hospital in Friedrichshain, Clinic of Internal Medicine – Angiology, Hemostasiology and Pulmonology, Berlin	Dr Robert Klamroth
**Greece**	"LAIKO" General Hospital, Blood Unit – National Reference Centre for Congenital Bleeding Disorders, Athens	Dr Olga Katsarou
**Hungary**	Heim Pal Children's Hospital, Department of Oncohematology, Budapest	Dr Marianna Zombori
	Medical Centre, Hungarian Defense Forces, National Hemophilia Center, Budapest	Dr Laszlo Nemes
	University of Debrecen Clinical Center, Institute of Internal Medicine, Debrecen	Dr Zoltan Boda
**Italy**	ULSS (Local Social Health Unit) n. 8 "Berica," Complex Operative Unit of Hematology, Vicenza	Dr Alberto Tosetto
	Maggiore Polyclinic Hospital, IRCCS Ca' Granda, General Medicine Hemostasis and Thrombosis, Milan	Dr Flora Peyvandi
**Japan**	Tokyo Medical University Hospital, Department of Laboratory Medicine, Tokyo	Dr Kagehiro Amano
	Nara Medical University Hospital, Department of Pediatrics, Nara	Dr Keiji Nogami
	Hospital of the University of Occupational and Environmental Health, Department of Pediatrics, Fukuoka	Dr Takuma Ito
	Ogikubo Hospital, Department of Blood Coagulation, Tokyo	Dr Azusa Nagao
	Nagoya University Hospital, Department of Blood Transfusion Service, Nagoya	Dr Nobuaki Suzuki
	St. Marianna University School of Medicine Hospital, Department of Pediatrics, Kanagawa	Dr Masashi Taki

(*Continued*)

 © 2025. The Author(s).

Supplementary Table S1 (*Continued*)

Country	Enrolling site	Principal investigator
**Mexico**	"Dr. Federico Gomez" Children's Hospital, Federal District	Dr Aida Moreno Gonzalez
	Clinical Research Center Chapultepec, S.A. de C.V., Michoacan	Dr Gregorio Campos Cabrera
	Mexico General Hospital, Mexico City	Dr Carlos Martinez Murillo
	Applied Neuroscience Research Institute A.C., Durango	Dr Sara Flores Tapia
**The Netherlands**	UMC Utrecht, Division of Internal Medicine and Dermatology, Utrecht	Dr Kathelijn Fischer
**Taiwan**	Changhua Christian Hospital, Changhua	Dr Ming-Ching Shen
	National Taiwan University Hospital, Taipei	Dr Sheng-Chieh Chou
**United Kingdom**	Royal Free Hospital, London	Dr Pratima Chowdary
	Basingstoke and North Hampshire Hospital, Hampshire	Dr Sarah Mangles
**United States of America**	University of Colorado Hemophilia and Thrombosis Center, Aurora, Colorado	Dr Michael Wang
	Bloodworks Northwest, Seattle, Washington	Dr Barbara Konkle
	East Carolina University, Department of Internal Medicine, Division of Hematology/Oncology, Greenville, North Carolina	Dr Darla Liles
	Hemostasis and Thrombosis Center of Nevada, Las Vegas, Nevada	Dr Amber Federizo
	Michigan State University Center for Bleeding and Clotting Disorders, East Lansing, Michigan	Dr Roshni Kulkarni
	University of California San Diego, Hemophilia & Thrombosis Treatment Center, San Diego, California	Dr Annette von Drygalski
	University of Florida Health (UF Health), Shands Hospital, Gainesville, Florida	Dr Tung Wynn

Supplementary Table S2 Bleed treatment

	Treatment regimen		
	Prophylaxis ($n = 139$)	On demand ($n = 19$)	Overall ($N = 157$)
**No. of injections required for bleed resolution[a]**			
Number of bleeding episodes, *n*<br>Median, *n* (Q1, Q3)<br>Mean, *n* (SD)	536<br>1.0 (1.0, 1.0)<br>1.9 (5.1)[b]	442<br>1.0 (1.0, 1.0)<br>1.3 (0.6)	978<br>1.0 (1.0, 1.0)<br>1.6 (3.8)[b]
**Total dose required for bleed resolution[a]**			
Number of bleeding episodes, *n*<br>Median, IU/kg (Q1, Q3)	525<br>37.1 (26.4, 50.8)	441<br>26.3 (17.0, 30.3)	996<br>30.2 (23.3, 40.7)
**Number of injections required for resolution of a bleeding episode[a]**			
1<br>2<br>3<br>4<br>>4	414 (77.2)<br>69 (12.9)<br>26 (4.9)<br>10 (1.9)<br>17 (3.2)	360 (81.4)<br>61 (13.8)<br>16 (3.6)<br>2 (0.5)<br>3 (0.7)	774 (79.1)<br>130 (13.3)<br>42 (4.3)<br>12 (1.2)<br>20 (2.0)

Abbreviations: ePD, electronic patient diary; Q, quarter; SD, standard deviation.
Notes: [a]Per bleeding event.
[b]The higher mean values were largely driven by 3 subjects in the prophylaxis regimen who had reported bleeding episodes with a high number of injections (Patient 1: 65, 46, and 24 injections for 3 reported bleeds; Patient 2: 71 injections; Patient 3: 47 injections). A subset of patients in the study received retraining on correct use of the ePD.

 © 2025. The Author(s).

Supplementary Table S3 Treatment-emergent adverse events

	Treatment regimen			
n (%)	Prophylaxis (*n* = 50)	On demand (*n* = 1)	Major surgery period (*n* = 2)	Overall (*N* = 51)
**Total number of TEAEs**	55	0	3	58
**Patients with ≥1 TEAE**	29 (58.0)	0	2 (100)	29 (56.9)
**Patients with ≥1 TESAE**	5 (10.0)	0	1 (50.0)	5 (9.8)
**Patients with ≥1 related TEAE or TESAE, *n* (%)**	0	0	0	0
**TEAEs leading to death[a]**	1 (2.0)	0	0	1 (2.0)
**TEAEs leading to study withdrawal[a]**	1 (2.0)	0	0	1 (2.0)
**Most common TEAEs[b]**				
Nasopharyngitis	5 (10.0)	0	-	5 (9.8)
Fall	4 (8.0)	0	-	4 (7.8)
Arthralgia	2 (4.0)	0	-	2 (3.9)
Back pain	2 (4.0)	0	-	2 (3.9)
Cough	2 (4.0)	0	-	2 (3.9)
Influenza-like illness	2 (4.0)	0	-	2 (3.9)
Upper respiratory tract infection	2 (4.0)	0	-	2 (3.9)

Abbreviations: AE, adverse event; TEAE, treatment-emergent adverse event; TESAE, treatment-emergent serious adverse event.
Notes: Percentages are based on the number of patients in the safety analysis set. Safety was assessed in a subset of the full analysis set, which included all patients who received rFVIIIFc (prophylaxis or on demand) as standard of care at any point in the study. Patients are included in each treatment regimen they participated in and, as such, may appear in more than one treatment regimen. Each patient is counted only once in the overall column. TEAEs are included in the treatment regimen coinciding with the onset date. AEs with missing causality assessment are included in the related TEAE or related TESAE.
[a]One patient in the prophylaxis group experienced a TEAE with fatal outcome (post-procedural haemorrhage following removal of a duodenal tumour).
[b]Experienced in >3% of patients overall.

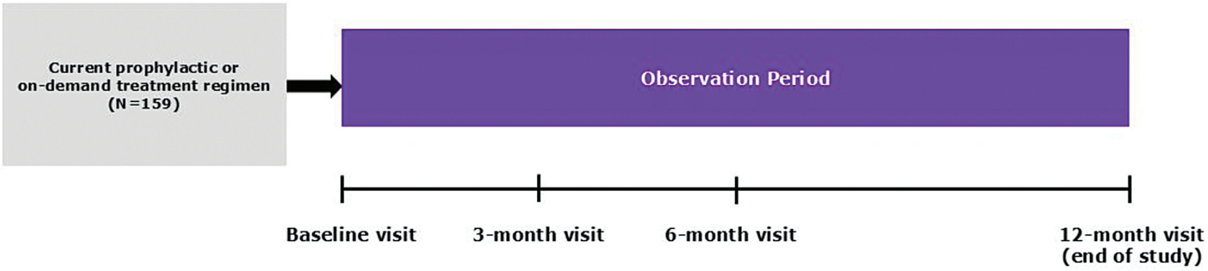

Supplementary Fig. S1 Study design.[a]
[a]Subjects were followed for up to 12 months. Subjects could withdraw early from the study to enter any subsequent Bioverativ-sponsored interventional study. In addition to the specified study visits at baseline and months 3, 6, and 12, subjects could attend their routine standard-of-care and as-needed visits.

 © 2025. The Author(s).

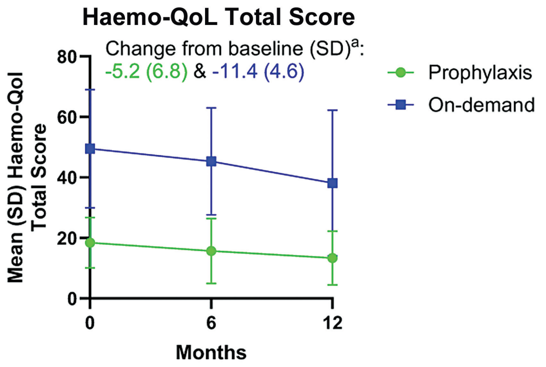

Number	Prophylaxis	On-demand
0 months	24	2
6 months	25	2
12 months	26	2
Change from baseline at 12 months	23	2

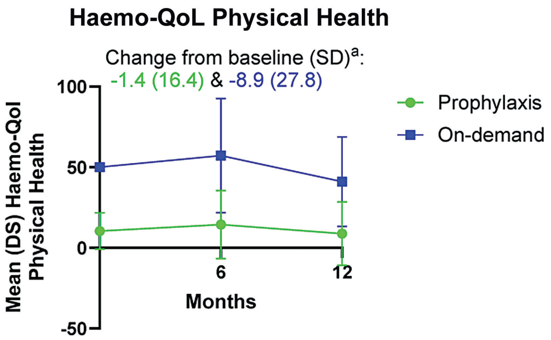

Number	Prophylaxis	On-demand
0 months	24	2
6 months	25	2
12 months	26	2
Change from baseline at 12 months	23	2

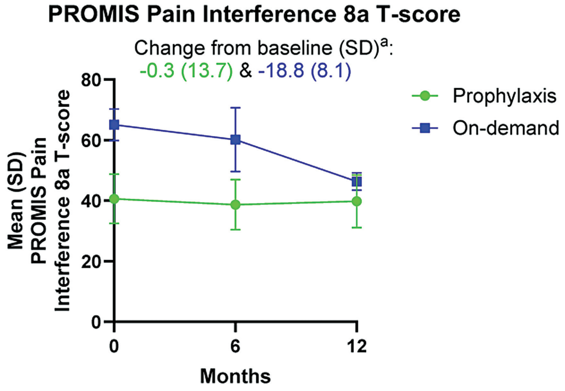

Number	Prophylaxis	On-demand
0 months	18	2
6 months	20	2
12 months	20	2
Change from baseline at 12 months	16	2

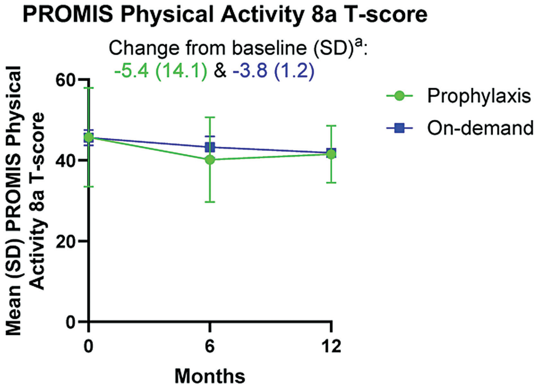

Number	Prophylaxis	On-demand
0 months	9	2
6 months	16	2
12 months	20	2
Change from baseline at 12 months	9	2

Supplementary Fig. S2 Haemo-QoL[a] and PROMIS pain interference[b] and physical activity[c] outcomes in paediatric participants, prophylaxis or on-demand regimens.

[a]Subscale scores and total scores are presented as Transformed Scale Scores (TSS) ranging from 0 to 100%; lower TSS indicates better quality of life. [b]Scores are presented as standardized T-scores, with lower scores indicating better health outcomes. [c]Scores are presented as standardized T-scores, with higher scores indicating better health outcomes. Abbreviations: Haemo-QoL, Haemophilia Quality of Life Questionnaire for Children and their Parents; PROMIS, Patient-Reported Outcomes Measurement Information System; SD, standard deviation.

 © 2025. The Author(s).
